# An Address

**Published:** 1867-01

**Authors:** G. A. Mills


### AN ADDRESS.

BY G. A. MILLS.

Read before the Brooklyn Dental Association, October 31, 1866.

Mr. President and Gentlemen:—You have assigned to me a subject, be it rightly delineated, I feel that I am wholly unable to master, and yet I may be able to present some thoughts for the consideration of the evening that will awaken new and valuable reflections, that we may carry out with us into the unknown future of our professional lives, and as we ascend the high hill of progression in the distant day (we may), *some one of us*, look down from the dizzy heights, which, step by step, we have gained, and rejoice that our lines were drawn in such pleasant places, that we have had so goodly a heritage as the Brooklyn Dental Association. Week after week, month after month, and year after year, has found us a band of brothers, faithful (in the main), to the trust that has been committed to us; uniting, and reuniting, those through kind bonds of brotherly love, casting our bread upon the waters in sanguine hopes to gather after many days, adding from time to time new accessions to our works until now, and to-night we *can* boast with holy pride of as worthy a prestige as any Dental Association of our much loved America. Now my brothers, with this escutcheon of fair fame, we are called to discuss the subject of Dental Ethics. What does the term mean? Ethics is the *science* of *duty*. *Science* is *knowledge*. Knowledge is clear perception. Now here, Dental Ethics, means (as applied to our profession), a clear perception of duty—to our patients—to each other—to ourselves and to God, and then let me say, it is not in our power to do our whole duty, as referred to above, if we do not recognize our obligations to the giver of all things. Man (and of course the Dentist), left to himself is influenced by a great variety of motives, aside from right, viz.: love of money, reputation, &c., &c. There are "many men of many minds, many men of many kinds," and, therefore, many reasons (so-called), for many Dentists doing as *they do*, and it will be claimed, that it is impossible to bind all to a code of laws. We are living in a time when the line of right is being more closely drawn toward the center of justice; and happy is that man who does not look through a glass darkly. How often the beauty of a sunset is hid from our vision by a dark and frowning cloud, we get glimpses of its glory out on its edge. With our profession, it has had a life of struggle, far back in the past some earnest soul caught glimpses of its future, and induced by the love of duty, sowed the seed in faith, and though cast about in the sturdy ship of progression, sometimes in winds, then for a time in calm, resting to trim its sails to gather fresh breezes and thus pressing it on with new hopes of reaching new fields wherein to sow fresher seeds and gather new fruits, the products, always, of the persistent labor of love, and behold to-night the fellowship of many tempests, her streamers of victory floating out from many a masthead, with many of her weather-beaten commanders still at the helm, giving a "certain sound" in command, surrounded by as brave a crew as the world ever saw. Is there one of us *now* that will forsake his post and turn back? No, no, let us from this time and forever buckle on more closely the insignia of our profession, take upon our lips and place upon the crests of our hearts, that brilliant watch-word, *duty*. Yes, *duty* everywhere, duty under all circumstances, cost what it may. Let us no longer be stimulated by that slimy lizzard of avarice, nor drink from that unsatisfying and uncertain fountain, *reputation*. But go from this place fixed in our determination, that the cases committed to our care henceforth and forever, *shall* receive from us our whole ability, dealt out in kindness, be they simple or complicated in their nature, daring not to trifle with those temples (indwellings of the ever-living soul). Whatever our hands find to do, let us do it with our might. Our *duty is* a specialty, and those patients that commit themselves to our care, are not in the aggregate, supposed to be familiar with the knowledge we *should* possess, therefore let us make no assumptions, but simply and purely, with a fervent desire to rightly instruct, to affilliate the confidence of each other, seeking unity, for "in union there is strength." This gained, a great point is gained toward the end sought. Prove to them the difference between *guessing* and *knowing*, in this you *will* have your reward. That we may always be ready to give a reason for the opinion we offer, or the diagnosis we make, it is absolutely necessary that we should have our bodies in keeping with the truth, thus we must be free from all those contaminating habits that *war* against the flesh, and necessarily weaken the intellect, misguide the motive power of our hands, and the *ideal*, we should desire, being first conceived in the mind, then wrought out by the fingers, we are in after life compelled to look with shame upon the wretched results of our labors. Do we ask, is it I? I answer, who of us is free? Let *love* be ever the mainspring of our motives, putting forth every effort to remove the *debris*, always found along the banks of every stream; for that which is worthless or weak, with no energy to guide, more often seeks the still and shallow water, and yet there are many who are found out in the swift current willing to risk their *puny lives* to gain a *hollow name*, often secured at the expense of their neighbors, by their valuable contributions, scattered wide and free, and seizing, as they do, from time to time, those little episodes of light, they venture out to the calm and placid waters of ease, and float here and there, and then with lazy flight seeking more comfort they cautiously alight like the crow; feast hurriedly upon the *scattered* and *growing seed*, and at the approach of him who sows, he hastily bestirs himself to gather his worthless carcase, flaps his wings away to yonder tree, sits and *caws*, as though he was some proud monarch of his time. Do we miss him by anything he has done? He simply leaves behind a few shapeless scratches, and we are told that he has departed. There are those who are weak and feel themselves to be so, to such we should always lend a helping hand, for, "as the twig is bent, the tree is inclined."

In our department let us show ourselves exemplary in all our dealings. By experience may we beget patience, which is a princely virtue to be possessed by us. It is said, "he that maketh haste to be rich is not just." How often do we see this truth vindicated by the advertisements of various kinds resorted to by members of our profession, giving guarantees which is not in the power of man to fulfill. Purchasing the good will of this and that M.D. and D.D., putting them (after) under the unrighteous obligation of saying that Dr. so and so is the finest Dentist in the land. When he is often known to be far from being any such thing. By this, progress is often hindered. If you know your ability, assert, and leave no stone unturned to prove it, for by your fruits you will be known; vindicate your professional brother as far as he is right, and should you be inquired of, regarding his claims for compensation for services rendered, act well your part. Are you familiar with his standard of character and practice. You are able to be true to your duty. You need not err, gentlemen, this precious word *duty* interweaves itself so tenaciously into all the intricacies of our daily life, we need to cherish it as a sweet morsel under the tongue. Follow its blessed teachings, and *all* these various evils we are called to denounce, by the adoption of the code of Ethics which is to be presented to you by the American Dental Association, will eradicate all necessities of this. Feeling that I may have already trespassed upon the time of our meeting, I leave the subject for your further consideration.

